# Characterizations of White Mulberry, Sea-Buckthorn, Garlic, Lily of the Valley, Motherwort, and Hawthorn as Potential Candidates for Managing Cardiovascular Disease—In Vitro and Ex Vivo Animal Studies

**DOI:** 10.3390/nu16091313

**Published:** 2024-04-27

**Authors:** Aleksandra Witkowska, Anna Gryn-Rynko, Patrycja Syrkiewicz, Klaudia Kitala-Tańska, Michał S. Majewski

**Affiliations:** Department of Pharmacology and Toxicology, Faculty of Medicine, University of Warmia and Mazury, 10-082 Olsztyn, Poland; aleksandra.witkowska@uwm.edu.pl (A.W.); anna.gryn-rynko@uwm.edu.pl (A.G.-R.); p.syrkiewicz@wp.pl (P.S.); klaudia.kitala@wp.pl (K.K.-T.)

**Keywords:** white mulberry, sea-buckthorn, garlic, lily of the valley, motherwort, hawthorn

## Abstract

Cardiovascular diseases are a broadly understood concept focusing on vascular and heart dysfunction. Lack of physical exercise, type 2 diabetes, obesity, hypertension, dyslipidemia, thromboembolism, and kidney and lung diseases all contribute to the development of heart and blood vessel dysfunction. Although effective and important, traditional treatment with diuretics, statins, beta blockers, calcium inhibitors, ACE inhibitors, and anti-platelet drugs remains a second-line treatment after dietary interventions and lifestyle changes. Scientists worldwide are still looking for an herbal product that would be effective and free from side effects, either taken together with or before the standard pharmacological intervention. Such herbal-originated medication therapy may include *Morus alba* L. (white mulberry), *Elaeagnus rhamnoides* (L.) A. Nelson (sea-buckthorn), *Allium sativum* L. (garlic), *Convallaria majalis* L. (lily of the valley), *Leonurus cardiaca* L. (motherwort), and *Crataegus* spp. (hawthorn). Valuable herbal raw materials include leaves, fruits, seeds, and even thorns. This short review focuses on six herbs that can constitute an interesting and potential therapeutic option in the management of cardiovascular disorders.

## 1. Introduction

**Figure 1:**
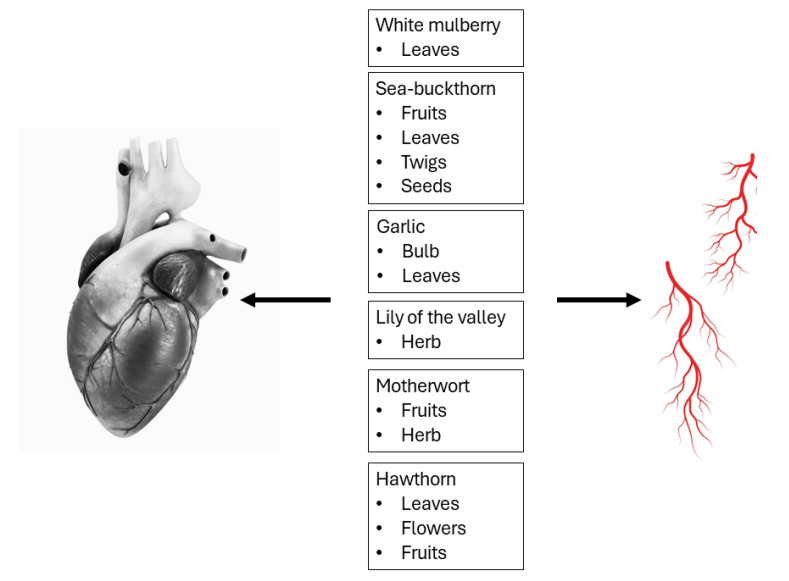
White mulberry, sea-buckthorn, garlic, lily of the valley, motherwort, and hawthorn in the prevention and treatment of cardiovascular disease.

As, in some cases, traditional pharmacological treatment in the management of cardiovascular disorders may fail, attention has focused on herbal medications to replace pharmacological treatment, especially for those who do not meet the criteria for typical pharmacological interventions. In this review, we focus on white mulberry, sea-buckthorn, garlic, lily of the valley, motherwort, and hawthorn, suggesting that these traditional herbs could contribute to the treatment of cardiovascular disorders. This review highlights the importance of herbal-originated medication, dividing it into different fractions, including leaves, fruits, seeds, and even thorns, which differ among themselves in terms of the content of active substances, as supported by the latest research. Our comprehensive literature search of the published literature encompassed various scientific databases, including PubMed, Scopus, Web of Science, and the Cochrane Library. We employed a wide range of keywords during this analysis, including “metabolic diseases”, “hypertension”, and “oxidative stress”, to capture the diverse uses of the six plants described here (Figure 1) and of their parts such as “leaves”, “fruits”, “seeds”, and “thorns” in “animal studies” both “in vitro” and “in vivo”/“ex vivo’’.

## 2. White Mulberry

*Morus alba* L. is a medium-sized deciduous tree which belongs to the *Moraceae* family and originates from and is widely cultivated in Asian countries. White mulberry is highly adaptable to different soil, climatic, and topographical conditions and is widely distributed from temperate to subtropical regions [1,2]. Mulberry leaves are a precious source of bioactive compounds, including flavonoids, phenolic acids, iminosugars, vitamins, organic acids, proteins, and macro- and micronutrients [3,4,5,6,7,8]. Because of their rich phytochemistry, the leaves exhibit a wide spectrum of pharmacological activities, such as antidiabetic, antibacterial, anticancer, hypolipidemic, antioxidant, antiatherogenic, and anti-inflammatory properties [2,3,5,6,7,8].

Furthermore, according to studies designed and conducted on animal models, white mulberry leaves are also considered an effective alternative therapy for cardiovascular diseases (CVDs), including coronary heart disease, arterial hypertension, prevention and/or treatment of myocardial infarction, and stroke [1,9,10,11,12,13]. Madhumitha and Indhuleka [9] as well as Nade et al. [10] reported that the plant extract may exert a beneficial role in the treatment of myocardial infarction and hypertension through the regulation of antioxidant defensive mechanisms. Male albino Wistar rats with induced myocardial infarction treated with the methanolic extract of white mulberry leaves experienced significantly increased activities of antioxidants such as superoxide dismutase (SOD), catalase (CAT), glutathione peroxidase (GPx), and glutathione–S–transferase (GSH) as well as a notable inhibition of the lipid peroxidative process [9,10]. Moreover, biochemical markers such as lactate dehydrogenase (LDH) and creatine kinase (CK) were significantly increased. The oral administration of mulberry extract significantly reduced heart rate, arterial pressure, the pressure-rate index, and heart weight [10]. In histopathological studies, the plant extract also contributed to the reconstruction of damaged fragments of the cardiac muscle, as well as to a reduction in the area of myocardial necrosis and its severity [9,10]. According to the authors, phenolic compounds are responsible for the above-mentioned properties of white mulberry leaves [9,10]. In agreement with these findings are Cao et al. [14], who explored the myocardial protective effects of the components of mulberry leaves in diabetic mice. Mulberry extract and the iminosugar 1-deoxynojirimycin (1–DNJ) significantly improved myocardial oxidative stress and fibrosis in the hearts of diabetic mice and consequently improved cardiac function [14]. Moreover, the authors found that 1–DNJ crucially improved the level of oxidative stress and inhibited the TGF–β–Smad2/3 pathway, ultimately ameliorating myocardial fibrosis and cardiac dysfunction. The authors concluded that 1–DNJ may be the main component of the plant extract that contributes to a protective effect on the diabetic myocardium [14]. Mulberry extract is also a promising candidate for an antiplatelet and antithrombotic agent [12]. In in vitro assays using isolated rat platelets, the plant extract showed a significant dose-dependent inhibition of collagen-induced platelet aggregation through reducing [Ca^2+^]i, ATP, and integrin αIIbβ3. Mulberry also attenuated serotonin secretion and thromboxane A_2_ formation. In addition, mulberry leaves at 100, 200, and 400 mg/kg significantly and dose-dependently attenuated thrombus formation in an in vivo rat arterio-venous shunt model [12]. Mulberry leaves may also become a useful drug for the treatment of metabolic syndrome and its related atherosclerotic lesion formation [15]. Yang et al. determined the effect of mulberry leaf extract and its major component, neochlorogenic acid, on the proliferation and migration of rat aortic vascular smooth muscle cells (VSMCs, A7r5 cell line) under diabetic cultured conditions [15]. Their results indicate the anti-atherosclerotic effects of plant extract and neochlorogenic acid in reducing vascular smooth muscle cell migration and proliferation under diabetic cultured conditions via inhibition of focal adhesion kinase (FAK) and small GTPase proteins, phosphoinositide 3–kinases (PI3K)/protein kinase B (Akt), and Ras-related signaling [15]. Meanwhile, Grassi et al. suggested that mulberry leaves are effective in improving endothelial function and that flavonoids, as fundamental vasoactive compounds, are responsible for the above-mentioned effect. The benefits of mulberry leaves on endothelial function were also seen by Carrizzo et al. [11]. In their vascular reactivity studies, these authors demonstrated that white mulberry extract evokes endothelial vasorelaxation through a nitric oxide (NO)-dependent pathway. They indicated that the vascular complex PERK/HSP90/eNOS is crucial for mulberry extract’s vasorelaxant action. Their molecular analysis highlighted an increase in endothelial nitric oxide synthase (eNOS) phosphorylation. Finally, they found that a single oral dose of white mulberry extract reduced blood pressure levels in vivo. This hypotensive effect started 2 days after the oral application of a single dose of the plant extract and after 4 days, blood pressure levels returned to the baseline, and the administration of a second dose evoked the same hypotensive effect as previously observed [11]. The cardiovascular effects of *Morus alba* L. (white mulberry) are presented in Table 1 and Table 2.

## 3. Sea-Buckthorn

*Elaeagnus rhamnoides* (L.) A. Nelson is a shrub which can reach the size of a small tree and belongs to *Elaeagnaceae* family. In folk medicine, sea-buckthorn has been used in the treatment of ulcers, wounds, inflammation, edema, and hypertension. Sea-buckthorn fruits (berries) and leaves constitute a well-known source of phytochemicals and have been widely explored, contrary to its seeds, which have been poorly studied as of yet. Sea-buckthorn fruits contain polyphenols (proanthocyanidins), flavonoids (flavonol glycosides isorhamnetin 3–O–hexoside–deoxyhexoside and isorhamnetin 3–O–hexoside), phenolic acids, vitamins (vitamin C), fatty acids, and phytosterols. Moreover, the fruits are a rich source of different secondary metabolites, i.e., triterpenes and triterpene derivates [16]. In another study, eleven flavonols were detected: six compounds derived from isorhamnetin, four compounds derived from quercetin, and kaempferol [17]. Sea-buckthorn leaf extract contains ellagitannins (mainly casuarinin, hippophaenin B, casuarictin, stachyurin, and strictinin or their isomers) as well as ellagic acid and its glycosides [16]. Flavonoids, including glycosides of isorhamnetin, kaempferol, and quercetin, are another component of the leaf extract [16]. The twig extract consists mainly of proanthocyanidins and catechin [16]. The seeds contain flavonoids, mostly glycosides of isorhamnetin, kaempferol, and quercetin. Smaller amounts of proanthocyanidins and catechin, triterpenoid saponins, and several unidentified polar and hydrophobic compounds have also been detected [18].

The consumption of sea-buckthorn flavonoids is inversely associated with increased mortality due to CVDs. These compounds may inhibit blood platelet activation by way of various mechanisms, including the GPIIb/IIIa-mediated signaling pathway, inhibiting the expression of COX, and regulating protein kinase C [16]. Triterpenes and their derivates present in sea-buckthorn fruits not only possess antioxidant properties but may also display anticoagulant attributes [19]. Sea-buckthorn fruits lower cholesterol concentration and inhibit blood platelet activation [19]. H–flavone extracted from the fruits and leaves effectively inhibited macrophage foaming and inflammation and reduced the risk of vascular plaque formation by upregulating the expression of CTRP6, a member of the C1q/TNF-related protein (CTRP) family. H–flavone may be used to treat and prevent atherosclerosis based on its substantial anti-inflammatory and hypolipidemic effects [20]. In another study, it was observed that the extract from sea-buckthorn leaves had a stronger antiplatelet potential than the extract from twigs [16]. However, both the leaf and twig extracts possess anti-platelet and anticoagulant properties [16]. The authors concluded that the leaf extract can be used in the prevention and treatment of CVDs associated with blood platelet hyperactivity [16]. Ethanolic seed and root extracts are better radical scavengers than leaf and stem extracts, which correlates with the presence of phenolic compounds in the active fractions [21]. Sea-buckthorn seeds possess anticoagulant potential and antioxidant activity that is not impaired by thermal processing, but more research is needed in order to ascertain which compounds are responsible for these effects, especially in an in vivo model [18].

In type 2 diabetes mellitus, sea-buckthorn berries suppressed hyperglycemia and decreased water intake in Zucker diabetic fatty rats [22]. In another study on mitigating induced oxidative stress in hyperlipidemic rats, sea-buckthorn berries diminished the oxidative stress in the heart, liver, and kidney [23]. In young male Sprague–Dawley (SD) rats, supplementation with sea-buckthorn berries (7–28 mg/kg) significantly improved the tolerance for hyperlipidemia, prevented endothelial dysfunction of the aorta by enhancing the activity of antioxidant enzymes, attenuating the levels of inflammatory cytokines such as TNF–α and IL–6, and decreased the level of eNOS, ICAM–1, and LOX–1 expression [24]. Sea-buckthorn leaves induced protection against hexachlorocyclohexane-induced oxidative stress in rats [25]. In Wistar rats, sea-buckthorn leaf extract (at 100 mg/kg body weight per os) protected against hepatic damage caused by lead acetate in drinking water [26]. In the livers of Sprague–Dawley rats, the phenolic-rich fraction from sea-buckthorn leaves prevented oxidative damage to major biomolecules and provided significant protection against carbon tetrachloride-induced oxidative damage [27]. Leaf aqueous extracts (200 and 800 mg/kg body weight) enhanced exercise capacity and protected against oxidative damage caused by exhaustive exercise in Wistar male rats [28]. The cardiovascular effects of *Elaeagnus rhamnoides* (L.) A. Nelson (sea-buckthorn) are presented in Table 3.

## 4. Garlic

*Allium sativum* L. belongs to the *Amaryllidaceae* family. The phytochemicals in garlic have a positive effect on the progression of hypertension, dyslipidemia, and atherosclerosis thanks to their anti-inflammatory and antioxidant properties, which are important in CVDs. The main bioactive chemical compounds in garlic are organosulfur compounds, which include alliin, allicin, E–ajoene, Z–ajoene, 2–vinyl–4H–1,3–dithiin, diallyl sulfide (DAS), diallyl disulfide (DADS), diallyl trisulfide (DATS), and allyl methyl sulfide (AMS) [30,31]. Alliin could become the precursor to all the bioactive compounds in garlic. Alliin is hydrolyzed by the enzyme alliinase to dehydroalanine and allyl sulfenic acid. As a result of biochemical changes, two molecules of allyl sulfenic acid combine to form allicin [32]. In subsequent spontaneous reactions, allicin can be spontaneously transformed into allyl polysulfides from thiosulfinates [33].

According to scientific reports, alliin is one of the compounds that lowers blood triglycerides and increases high-density lipoprotein (HDL) levels [34,35]. Alliin also prevents myocardial tissue lipid accumulation in rats with isoproterenol-induced myocardial ischemia. This is probably due to its free-radical-scavenging effect. It also helps lower lipid levels by reducing or inhibiting the process of lipid peroxidation [36]. Allicin has a similar mechanism of action to alliin. This includes an anti-oxidative effect, which improves mitochondrial functioning, affecting their integrity and longevity. It may be beneficial in CVDs associated with mitochondrial dysfunction. Allicin advantageously affects CVD risk factors (dyslipidemia, obesity, atherosclerosis, oxidative stress, hypertension, diabetic cardiomyopathy, arrhythmias, and myocardial hypertrophy) by regulating many intracellular signaling pathways and thermogenic genes. Some reports also stated that allicin inhibits vascular calcification, which affects the elasticity of vessels and thus the deposition of atherosclerotic plaques [37,38]. Furthermore, allicin improves endothelial progenitor cell migration [39]. Allicin decreased blood pressure and plasma triglyceride levels in hypertensive rats [40]. 2–Vinyl–4H–1,3–dithiin is another compound of the metabolic pathway of allicin, with anti-proliferative and anti-immigration effects, which protects vascular smooth muscle cells against reactive oxygen species induced by angiotensin II [41]. Another compound, AMS, reduces fetal gene expression and oxidative stress, which has a positive effect on myocardial fibrosis and hypertrophy. In addition, AMS has a similar effect on the mitochondria as allicin, and it improves the rate of oxygen consumption, production of reactive oxygen species, and membrane potential [42,43]. Tsai et al. [44] found that DATS increases the expression of proteins in the IGF1R survival signaling pathway, which contributes to the cardioprotective effect. DATS also induces an antioxidant effect on processes in the mitochondria, protecting the vascular endothelium damaged by hyperglycemia [45]. Lestari et al. [46] proved that the organosulfur compounds in garlic inhibit the fatty acid synthase (FAS) enzyme and have the same binding site as statins. The FAS enzyme catalyzes the reaction of fatty acid synthesis, the inhibition of which reduces the level of lipids in the blood. In addition, the phytochemicals in garlic may inhibit platelet aggregation by increasing cAMP and cGMP levels and preventing the GPIIb/IIIa receptor from binding to fibrinogen. This may be helpful in anticoagulant therapy in people with CVDs [47,48].

Garlic may be helpful in the prevention and treatment of many CVDs; however, evidence-based medicine research is needed to standardize dosages and specific uses in humans. The phytochemicals in garlic can affect other systems and processes in the human body that directly or indirectly affect the cardiovascular system. The cardiovascular effects of *Allium sativum* L. (garlic) are presented in Table 4 and Table 5.

## 5. Lily of the Valley

*Convallaria majalis* L. is a species of perennial rhizome belonging to the *Asparagaceae* family. It grows in areas with a temperate climate, is distinguished by its resistance and durability, and is commonly found in deciduous forests in the Northern hemisphere [49]. Lily of the valley is a source of many bioactive compounds, such as glycosides [50]. This popular garden plant is also well known to be toxic for both humans and animals [51]. It has been in use since the 16th century in herbal medicine, nowadays mostly as a component of herbal extracts [52]. Due to its health-promoting properties, it is used as a cardioprotective agent [53]. Lily of the valley, when used in appropriate doses, is an effective alternative in the treatment of cardiac dysfunction [54].

The plant extract contains many toxins, such as around 40 cardiac glycosides, convallarin, convallotoxin, convalloside, convallasaponin, cholestane glycoside, strophanthidin, cannogenol, sarmentogenin, dipindogenin, hydroxysarmentogenin, and saponins [51]. Cardiac glycosides are capable of inhibiting the sodium–potassium pump (Na^+^/K^+^–ATPase) [52]. Positive inotropic effects are caused by raising the level of sodium ions in cardiac myocytes, which leads to an increase in the level of calcium ions, and as a result, the force of contraction of the heart increases [55]. The primary active glycoside with structural similarity to digoxin is convallatoxin [54]. As shown in studies, cardiotonic steroids, including convallatoxin, in therapeutic doses have a positive impact on Na^+^/K^+^–ATPase [56]. Those digitalis-like properties are responsible for the positive inotropic effect. The transformation of convalloside, the basic metabolic glycoside, into convallatoxin and other cardiac glycosides takes place in the plant [54]. Hoi et al. [57] confirmed the convallatoxin-induced positive inotropic effect. Moreover, convallatoxin has both vasoconstrictor and vasodilator effects. Furthermore, it affects cardiac stroke volume, pulse pressure, and cAMP activity [57]. Recent research has focused on Na^+^/K^+^–ATPase as a versatile signal transducer in which cardiac glycosides activate many signal transduction pathways and regulate important cellular processes such as cell growth, motility, and apoptosis. In addition, cardiac glycosides cause a positive inotropic effect with calcium at concentrations that do not interfere with the pumping activity of Na^+^/K^+^–ATPase. Cardiac glycosides may be beneficial in the treatment of cardiac diseases by activating the signaling properties of Na^+^/K^+^–ATPase and due to their beneficially enhanced inotropy-to-toxicity ratio [53]. Convallamaroside, a steroidal saponin isolated from lily of the valley, may inhibit angiogenesis and has anticancer activity [58]. Matsuo et al. [59] confirmed those findings by determining cytotoxic activity against tumor cells in many steroidal glycosides found in lily of the valley. Furthermore, lower mortality rates in patients with cancer receiving cardiac glycosides were noticed [60]. Moreover, lily of the valley was also found to inhibit lipoxygenase, an enzyme which takes part in arachidonic acid metabolism [61]. The cardiovascular effects of *Convallaria majalis* L. (lily of the valley) are presented in Table 6.

## 6. Motherwort

*Leonurus cardiaca* L. is an herbaceous perennial plant belonging to the *Lamiaceae* family. Motherwort is native to central Asia and southeastern Europe, but it is now found worldwide due to its use as a traditional herbal medicine [62]. According to the latest pharmacognostic and phytochemical studies, the herb contains various classes of secondary metabolites such as diterpene bitter principles (leocardin), labdane-type diterpenes (15–O–ethylleopersin C, 15–O–methylleopersin C, and 15–EPI–O–methylleopersin C, leocardine, leosibiricine), iridoide monoterpenes (ajugoside, ajugol, galiritoside, garpagide, reptoside), flavonoids (lavandulifolioside, verbascoside, rutin, quercitrin, isoquercitrin, hyperoside, genkwanin, kempferol, apigenin), alkaloids (leonurine, stachydrine), p–hydroxycinnamic acid derivatives (caffeic, chlorogenic, cichoric, ferulic, rosmarinic), tannins, cycloleonuripeptides (A, B, C, D), triterpenoids of the ursane class (ursolic acid, ilelatifol D, corosolic acid, euscaphic acid), and essential oils (α–humulene, α–pinene, β–pinene, linalool, limonene) [63,64,65,66,67,68,69,70]. Several pharmacological studies have confirmed that this class of secondary plant metabolites present in motherwort have an impact on a wide variety of biological activities, which include analgesic, antibacterial, anti-inflammatory, anti-oxidative, antihypertensive, neuroprotective, and sedative properties [66,67,68,69].

However, motherwort was initially traditionally used in clinical and medical practice as a herbal remedy regulating the heart’s activity rhythm, in angina pectoris, cardiovascular neuroses, tachycardia, and in the initial stages of hypertension [71,72]. Currently, the extract is frequently used as a meritorious medicinal resource to treat anxiety, sleeplessness, central nervous system and gynecological conditions, and CVDs [68,70,72,73]. In Poland, combination products containing motherwort have been used for 30 years and their consumption is recommended 2–3 times daily (1.5–2.5 g) as a sedative medication in nervous issues complaints. In Lithuania, motherwort-based medications in the form of single-ingredient or combination products are used for the primary prevention of CVDs and stress-, anxiety-, nervousness-, thyroid hyperfunction-, or climacteric-related heart dysfunction. In Germany, motherwort-based medications are used to support the function of the cardiovascular system. In Latvia, motherwort tincture is recommended for reducing mild nervous tension and in cases of functional heart disorders manifested as palpitations, intermission, or long-term pressure pain in the heart [74]. Nevertheless, the wide spectrum of motherwort’s medicinal properties related to CVDs is one of its most crucial and significant applications. Also, the European Medicines Agency (EMA) has recommended an extract obtained from motherwort as a potential drug [74]. Motherwort can improve endothelial dysfunction and cardiac function and may serve as a significant strategy to prevent the progression of pathological cardiac hypertrophy as well as ischemic heart diseases and hypertension [71,75,76,77,78]. Xie et al. reported that stachydrine, one of the major components of motherwort, possesses vascular-protective activities and can help minimize the risk of developing CVDs [75]. Their results revealed a new mechanism by which the natural alkaloids in motherwort effectively reversed homocysteine-induced endothelial dysfunction and prevented endothelial nitric oxide synthase (eNOS) uncoupling by increasing the expression of enzyme GTP–cyclohydrolase I (GTPCH1) and dihydrofolate reductase (DHFR) [75]. In agreement with those findings are Zhao et al. [75], who proved that stachydrine can be a promising candidate for treating cardiac hypertrophy. Other animal experiments revealed that alkaloids exhibit antioxidant and anti-inflammatory effects against isoproterenol-induced cardiac hypertrophy and fibrosis, mainly by inhibiting the NF–κB and JAK/STAT signaling pathways in rats. Moreover, stachydrine attenuated oxidative stress levels in the serum of isoproterenol-induced (ISO) cardiac hypertrophy rats, as shown by an increased activity of superoxide dismutase and decreased malondialdehyde level [76]. Additionally, Xu et al. [77] demonstrated that leonurine exhibited potent cardioprotective effects in a rat model of myocardial infarction by inducing anti-apoptotic effects by activating the PI3K/AKT/GSK3β signaling pathway, which promoted the expression of anti-apoptosis proteins, decreased the expression of pro-apoptosis proteins, and inhibited the activity of cleaved caspase3. The clinical trial conducted by Shikov et al. [71] on fifty patients treated for 28 days with 1200 mg motherwort oil extract revealed positive effects on arterial blood pressure in patients with stage 1 and 2 hypertension who were experiencing anxiety and sleep disorders. Side effects were minimal in all groups. Bernatoniene et al. [78] gave evidence of the cardioprotective effects of the extract of motherwort herb on cardiomyopathy. This effect was associated with the ability of the herb’s main components (chlorogenic acid, orientin, quercetin, hyperoside, and rutin) to partially inhibit (by ~40%) the mechanism of free radicals in mitochondria. The cardiovascular effects of *Leonurus cardiaca* L. (motherwort) are presented in Table 7 and Table 8.

## 7. Hawthorn

*Crataegus* spp. is a small-to-medium-sized tree and shrub which belongs to the *Rosaceae* family [79,80]. The hawthorn is predominantly found in the Northern hemisphere’s temperate regions, with substantial populations in North America, Europe, and Asia [81,82]. Hawthorn’s incorporation into traditional medicinal systems can be traced back to ancient times [83]. The Macedonians and another ancient cultures used hawthorn in their rituals, considering it a symbol of hope and happiness while concurrently recognizing its medicinal virtues [84]. In traditional Chinese medicine, Crataegus pinnatifida’s fruits, often referred to as Chinese hawthorn or shanzha, have been exploited over centuries to enhance digestion and improve blood circulation [85,86]. Traditional European medicine also emphasizes hawthorn’s medicinal importance. In the early 19th century, Irish doctors observed hawthorn’s effectiveness in treating heart conditions, which led to its subsequent incorporation into the British Herbal Pharmacopoeia [87,88]. The Crataegus genus boasts over 280 species [89], creating a broad spectrum of plant types with similar medicinal properties. The species most frequently utilized for medical purposes are *C. monogyna* and *C. laevigata* [90,91]. However, various species are used in different cultures and traditions depending on regional availability and specific uses [92].

Hawthorn’s rich phytochemical composition underpins its therapeutic potential, particularly concerning cardiovascular health [93]. The primary bioactive compounds in hawthorn include flavonoids, oligomeric procyanidins, and triterpene acids, all contributing protective effects on cardiac and vascular health [80,87]. Flavonoids are a class of polyphenols recognized for their potent antioxidant properties [94]. Vitexin, hyperoside, and rutin are flavonoids present throughout the hawthorn plant and have been associated with vasodilatory and anti-arrhythmic effects and the alteration of lipid profiles to lower lipid levels [95,96]. They influence the cardiovascular system by scavenging free radicals and reducing oxidative stress, thus protecting myocardial cells from oxidative damage [95]. Additionally, they help to reduce blood pressure and improve coronary artery blood flow by dilating blood vessels [96].

Oligomeric procyanidins, found predominantly in hawthorn leaves and flowers, enhance coronary blood flow and cardiac muscle contractions and exert a mild hypotensive effect, which may benefit heart failure treatment [97,98]. They are known to improve cardiac output and circulation by increasing the force of cardiac muscle contractions (positive inotropic effect) and mildly lowering blood pressure (hypotensive effect), enhancing cardiac efficiency overall [98,99].

The triterpene acids found in hawthorn fruit include ursolic and oleanolic acids. These anti-oxidative agents support the cardiovascular system by protecting against myocardial ischemia and atherosclerosis and decreasing inflammation [95,100]. They also inhibit apoptotic pathways, thus demonstrating a protective role against myocardial ischemia [100,101].

Beyond these primary bioactive compounds, the hawthorn plant is rich in sterols, amines, and vitamins, which may synergistically enhance its overall therapeutic efficacy [102]. In essence, the various bioactive compounds within hawthorn offer a multi-target approach preventing and treating CVDs. This comprehensive mode of action underscores the potential of hawthorn as a natural therapeutic agent in cardiovascular health.

Scientific research validates hawthorn’s efficacy in promoting cardiovascular well-being. There are an array of clinical trials affirming the beneficial potential of hawthorn in navigating conditions such as heart failure and hypertension, amongst other CVDs. A comprehensive meta-analysis spearheaded by Pittler et al. attests to the medicinal potential of hawthorn extract regarding chronic heart failure [87]. They delve into ten randomized clinical trials encompassing 855 patients, concluding that hawthorn extract brings about a marked increase in maximal workload, exercise tolerance, and ejection fraction. Notably, the application of this extract led to a significant reduction in the pressure-heart rate product—a crucial metric of cardiac workload. In the realm of hypertension, Walker et al. investigated hawthorn’s effects through a randomized controlled trial [103]. Upon treating the participants with hawthorn extract over ten weeks, the researchers noted a decrease in diastolic blood pressure, indicative of the hypotensive effect of hawthorn. Exploring hawthorn’s benefits in human heart failure, Zick et al. conducted a rigorous randomized trial [104]. Their findings underscored an improvement in the symptoms associated with heart failure alongside a significant enhancement in the quality of life of patients after undergoing treatment for 16 weeks. Another study conducted by Hanus et al. focused on the effects of administering hawthorn fruit extract to patients with heart failure [105]. They found that the treatment substantially improved patients’ physical capacity while alleviating symptoms such as shortness of breath and fatigue. However, the promising narrative spun around hawthorn is not devoid of the need for caution and further investigation. While current research lends credence to the therapeutic potential of hawthorn, it is necessary to execute more extensive, methodically designed clinical trials [90]. Progress in the field would aid in solidifying our understanding of hawthorn’s efficacy, determining the optimal dosage, and mapping out its safety profile while using it to treat CVDs. The cardiovascular effects of *Crataegus* spp. (hawthorn) are presented in Table 9.

## 8. Conclusions

The study of herbs and their medicinal significance in cardiovascular health is a rapidly expanding field. With continuing studies bolstering the therapeutic potential of herbs, numerous areas of intrigue call for extensive exploration. To begin with, existing research primarily concentrates on hawthorn’s efficacy in heart failure, especially NYHA class II heart failure. While the preliminary results are encouraging, the effectiveness of hawthorn in more advanced heart failure stages, namely NYHA classes III and IV, necessitates comprehensive examination [90]. Further, research to ascertain the benefits of herbs in other CVDs, including hypertension, atherosclerosis, and ischemic heart disease, would significantly extend its potential therapeutic applications. The mechanistic action of herbs, albeit partially comprehended, warrants additional scrutiny. Unraveling the molecular mechanisms underpinning their cardiovascular effects would validate its therapeutic use scientifically and facilitate the development of innovative drugs derived from herbal leaves, fruits, and seeds. Moreover, responses to herbs may vary significantly across individuals due to differences in genetics, age, gender, and the presence of co-morbidities. As such, future research endeavors should identify the factors that predict the therapeutic response to these herbs and adjust treatments accordingly. Although white mulberry, sea-buckthorn, garlic, and motherwort are generally perceived as safe, information on their long-term safety and side effect profiles remains scarce. More studies should investigate the safety of using these herbs during pregnancy and lactation, along with their interactions with other prevalent CVDs medications. We recommend exercising care when using hawthorn extract with other anticoagulant and/or antiplatelet drugs or undergoing surgery. Finally, clinical research should go beyond gauging these herbs’ efficacy and safety, incorporating patient-centered outcomes such as quality of life, patient satisfaction, and healthcare costs. Though initial data hint at these herbs’ potential to enhance the quality of life in heart failure patients, these results need to be confirmed through comprehensive, rigorously designed studies. While the evidence strongly backs these herbs’ therapeutic potential in cardiovascular medicine, numerous aspects demand further probing to exploit its medicinal virtues fully. Considering the global burden of CVDs, future research efforts that address these gaps could significantly improve cardiovascular health worldwide.

## Figures and Tables

**Table 1:** Cardiovascular effects of *Morus alba* L. (white mulberry), *Moraceae* family—ex vivo studies.

Herbal Raw Material	Active Compounds	Functions	Mechanism of Action	Model	Dose	References
Leaves	Phenolic groups (naringenin and quercetin)	Cardioprotective Antioxidant	↑ scavenging of free radicals (OH) ↓ peroxidation of lipids ↓ GSH, Gpx, SOD, CAT, TBARS	male albino Wistar rats	500 mg/kg b.w. for 15 days p.o.	[9]
	Flavonoids (isoflavones, flavanone, flavonols, morusin, cyclomorusin, and neocyclomorusin) Novel prenylated flavonoids (isoquercetin, quercetin, astragalin, and scopoline) Glycosides (namelyroseoside III and benzyl D–glucopyranoside)	Antihypertensive Antioxidant Cardioprotective	↑ cellular antioxidants (↑ GSH, SOD, and CAT, ↓ LPO) ↓ heart rate, ↓ heart weight ↓ pressure-rate index ↓ levels of cardiac damage marker enzymes	male Wistar rats	25, 50, 100 mg/kg b.w. for three weeks p.o.	[10]
		Antihypertensive Hypolipidemic Vascular improvement Lipid regulation agent	↓ of cell adhesion molecule (E–selectin, VCAM–1, ICAM–1) expression in the aorta	male rats	100, 200 mg/kg b.w. for 14 weeks	[13]
	Rutin, chlorogenic acid, astragalin	Vascular effect	↑ NO, eNOS ↑ phosphorylation of PERK and HSP90 at threonine	mice	100, 200, or 400 mg/kg b.w.	[11]
	Rutin and isoquercetin	Antiplatelet Antithrombotic Prevention or treatment of myocardial infarction	↓ MAPK–integrin α IIb β 3 ↓ PLA2/TXA_2_ routes ↓ TXB_2_ formation ↓ serotonin secretion ↓ aggregation ↓ thrombus formation	male Sprague–Dawley rats	100, 200, 400 mg/kg b.w. for 3 days	[12]
	Mulberroside A, chlorogenic acid, cryptochlorogenic acid, astragaloside, resveratrol, scopoletin, 1–deoxynojirimycin	Glucose improvement Lipid metabolism improvement	↓ TGF–β–Smad2/3	male C57BL/6 mice	1000 mg/kg b.w. for 12 weeks p.o.	[14]

↑—increased, ↓—decreased.

**Table 2:** Cardiovascular effects of *Morus alba* L. (white mulberry), *Moraceae* family—in vitro studies.

Herbal Raw Material	Active Compounds	Functions	Mechanism of Action	Model	Dose	References
	Neochlorogenic acid	Anti-atherosclerotic	↓ FAK/small GTPase proteins ↓ PI3K/Akt ↓ Ras-related signaling. ↓ vascular smooth muscle cell (VSMC) migration and proliferation	A7r5 cells (VSMCs)	3.0 mg/mL for 24 h	[15]

↑—increased, ↓—decreased

**Table 3:** Cardiovascular effects of *Elaeagnus rhamnoides* (L.) A. Nelson (sea-buckthorn), *Elaeagnaceae* family—in vitro studies.

Herbal Raw Material	Active Compounds	Functions	Mechanism of Action	Dose	References
Fruits	Polyphenols (proanthocyanidins) Flavonoids (flavonol glycosides isorhamnetin 3–O–hexoside–deoxyhexoside and isorhamnetin 3–O–hexoside) Phenolic acids Vitamins (vitamin C) Fatty acids and phytosterols Triterpenes and triterpene derivates (derived from isorhamnetin) Compounds derived from quercetin and kaempferol	Anti-platelet Anticoagulant	↓ surface exposition of GPIIb/IIIa and P–selectin	50 μg/mL; 10 min	[17,29]
Leaves	Ellagitannins (casuarinin, hippophaenin B, casuarictin, stachyurin, strictinin, or their isomers) Ellagic acid and its glycosides Flavonoids (glycosides of isorhamnetin, kaempferol, and quercetin)	Anti-platelet	↓ surface exposition of P–selectin ↓ surface exposition of GPIIb/IIIa active complex	5 and 50 μg/mL; 30 min	[16]
Twigs	Proanthocyanidins Catechin	Anticoagulant Anti-platelet	↓ surface exposition of P–selectin ↓ surface exposition of GPIIb/IIIa active complex	5 and 50 μg/mL; 30 min	[16]
Seeds	Flavonoids (glycosides of isorhamnetin, kaempferol, and quercetin) Proanthocyanidins and catechin, triterpenoid saponins, and several unidentified polar and hydrophobic compounds	Antioxidant Anticoagulant	↓ lipid peroxidation and protein carbonylation ↓ oxidation of thiol groups	0.5, 5.0, 50 µg/mL	[18]

↑—increased, ↓—decreased.

**Table 4:** Cardiovascular effects of *Allium sativum* L. (garlic), *Amaryllidaceae* family—ex vivo studies.

Herbal Raw Material	Active Compounds	Functions	Mechanism of Action	Model	Dose	References
Bulb	Organosulfur compounds: alliin, allicin, E–ajoene, Z–ajoene, 2–vinyl–4H–1,3–dithiin, diallyl sulfide (DAS), diallyl disulfide (DADS), diallyl trisulfide (DATS), and allyl methyl sulfide (AMS)	Hypoglycemic Hypolipidemic	↓ total triglycerides and free fatty acids ↑ HDL cholesterol	mice	0.1 mg/mL for 8 weeks Alliin (S–allyl cysteine sulfoxide) p.o	[34]
		Antioxidant	↓ lipid peroxidation ↓ free radicals	male albino Wistar rats	2 mL S–allyl cysteine sulfoxide (SACS) p.o.	[30,31,36]
		Antioxidant	↑ glutathione levels in vascular endothelial cells ↓ triglycerides	male SHR	80 mg/kg/day allicin p.o.	[40]
		Antioxidant	↓ cardiac hypertrophy markers	male Sprague–Dawley rats	250 mg/kg garlic	[42]
					25, 50, 100, 200 mg/kg/day Allylmethylsulfide (AMS) p.o.	[43]
		Antioxidant Vascular	ND ^1^	obese diabetic rats	5 mg/kg/day diallyl trisulfide (DAT)	[45]
		Lipid regulation	↓ lipogenesis and cholesterogenesis	male albino diabetic rats	1 mL garlic aqueous leaf extract	[35]

^1^ND—not determined. ↑—increased, ↓—decreased.

**Table 5:** Cardiovascular effects of *Allium sativum* L. (garlic)*, Amaryllidaceae* family—in vitro studies.

Herbal Raw Material	Active Compounds	Functions	Mechanism of Action	Model	Dose	References
Bulb	Organosulfur compounds	Antioxidant Vascular	↑ NO and eNOS	endothelial progenitor cells	100 µg/mL 200 µg/mL 400 µg/mL allicin	[39]
		Antioxidant	↓ ROS ↓ cell growth and migration	vascular smooth muscle cells (VSMCs)	10 and 100 μg/L 2–vinyl–4H–1,3–Dithiin	[41]
		Cardioprotective Anti-apoptotic	↑ IGF1R survival signaling pathway protein expression ↑ PI3K/Akt pathway ↓ ROS	cardiomyocyte cell line H9c2 and hearts from rats with streptozotocin-induced diabetes mellitus	10 μM diallyl trisulfide (DAT)	[44]
		Anti-aggregation	↓ platelet aggregation ↓ cAMP and cGMP stimulation ↓ fibrinogen binding to the GPIIb/IIIa receptor	human platelet aggregation	6.25% aged garlic extract	[47]

↑—increased, ↓—decreased.

**Table 6:** Cardiovascular effects of *Convallaria majalis* L. (lily of the valley), *Asparagaceae* family—in vitro studies.

Herbal Raw Material	Active Compounds	Functions	Mechanism of Action	Model	Dose	References
Herb	Cardiac glycosides: convallotoxin, convallarin, convalloside, convallasaponin, cholestane glycoside, strophanthidin, cannogenol, sarmentogenin, dipindogenin, hydroxysarmentogenin, saponins	Heart failure	↓ Na^+^/K^+^–ATPase inhibition ↑ Na^+^ ions ↑ Ca^2+^ ions positive inotropic effect ↓ viability of HUVECs ↑ TF mRNA and protein expression	serum human umbilical vein endothelial cells (HUVECs)	50 and 100 nM for 4 h	[51,52,55]
		Anti-inflammatory	↓ lipoxygenase inhibition	linoleic acid	5 g of the roots	[61]
	Convallatoxin/Convallaria keiskei	Cardiovascular effect	↑ atrial stroke volume and pulse pressure positive inotropic effect vasoconstrictor and vasodilator effects ↑ cAMP efflux ↑ K^+^ concentration	beating rabbit atria, male New Zealand white rabbits weighing about 2 kg	1 × 10^−5^ M Convallatoxin 3 × 10^−6^ g/mL Convallaria keiskei	[54,56,57]
	Convallamaroside	Anticancer	↓ angiogenesis	mice undergoing tumor angiogenesis reaction induced by tumor cells	10, 20, 50, 100 µg/day 1.5 h incubation	[58,59,60]

↑—increased, ↓—decreased.

**Table 7:** Cardiovascular effects of *Leonurus cardiaca* L. (motherwort), *Lamiaceae* family—ex vivo studies.

Herbal Raw Material	Active Compounds	Functions	Mechanism of Action	Model	Dose	References
ND ^1^	Stachydrine	Cardioprotective Antioxidant Anti-inflammatory	↓ NF–κB and JAK/STAT	male Sprague–Dawley rats	10 and 40 mg/kg b.w. for 21 days p.o.	[76]
Herb	Leonurine	Antiapoptotic Cardioprotective	↑ PI3K/AKT/GSK3β	male Sprague–Dawley rats	15 mg/ kg b.w. for 28 days p.o.	[77]
	Labdane diterpenes: leosibirin, leosibiricin, 19–acetoxypregaleopsin Flavonoid glycosides based on querectin and apigenin, phenylpropanoids: eugenol, lavandulifolioside, alkaloids: stachydrine, betonicine, leonurine, iridoids: ajugol, ajugoside, harpagide	Antihypertensive Psycho-neurological	ND ^1^	male and female patients	1200 mg per day for 28 days	[71]

^1^ND—not determined. ↑—increased, ↓—decreased.

**Table 8:** Cardiovascular effects of *Leonurus cardiaca* L. (motherwort)*, Lamiaceae* family—in vitro studies.

Herbal Raw Material	Active Compounds	Functions	Mechanism of Action	Model	Dose	References
Fruits	Stachydrine	Vasoprotective	↑ expression of PTCH1 and DHFR	isolated rat thoracic aortas, mesenteric arteries, and renal arteries from male Sprague–Dawley rats; bovine aorta endothelial cells (BAECs)	10 μM for 12 h; 0–10 μM for 24 h	[75]
Herb	Chlorogenic acid, orientin, quercetin, hyperoside, and rutin	Cardioprotective	↓ mitochondrial ROS generation	rat heart mitochondria	6.8 μg/mL 18.2 μg/mL	[78]

↑—increased, ↓—decreased.

**Table 9:** Cardiovascular effects of *Crataegus* spp. (hawthorn)*, Rosaceae* family—ex vivo studies.

Herbal Raw Material	Active Compounds	Functions	Mechanism of Action	Model	Dose	References
Leaves	Flavonoids (quercetin, vitexin–2″–O–α–L–rhamnoside, hispertin, pinnatifinosides I, cratenacin), oligomeric proanthocyanidins (proanthocyanidin A2)	Antioxidant Cardioprotective Antihypertensive Vasodilator	↑ myocardial perfusion ↓ of phosphodiesterase, ↑ in cGMP in smooth muscle cells	diabetic and non-diabetic rats	5 or 10 mg/kg	[106,107]
Flowers	Flavonoids (rutin, hyperoside, vitexin–2″–O–rhamnoside), phenols (chlorogenic acid), oligomeric proanthocyanidins (proanthocyanidin A2)	Antioxidant Cardioprotective Anti-inflammatory Anti-atherosclerotic Anticoagulant	↑ improving the blood flow in blood vessels	Sprague–Dawley rats	100, 200, and 500 mg/kg for 3 weeks p.o.	[106,108,109]
Fruits	Polyphenols, flavonoids (hyperoside, hesperidin, rutoside, crataequinone A–B), vitamin C, proanthocyanidins (procyanidin B2), phenols (chlorogenic acid)	Antioxidant Cardioprotective Hypolipidemic Anti-aggregation	ND ^1^	12-week-old rats 8-week-old SHR	1.08 g/kg body weight per day intragastrically 30 mg/kg Procyanidin	[106,108,110,111,112]

^1^ND—not determined. ↑—increased, ↓—decreased.

## Data Availability

Not applicable.
